# Pyroptosis-Related Gene Model Predicts Prognosis and Immune Microenvironment for Non-Small-Cell Lung Cancer

**DOI:** 10.1155/2022/1749111

**Published:** 2022-08-31

**Authors:** Lianxiang Luo, Xinyue Yao, Jing Xiang

**Affiliations:** ^1^The Marine Biomedical Research Institute, Guangdong Medical University, Zhanjiang, Guangdong, China 524023; ^2^The Marine Biomedical Research Institute of Guangdong Zhanjiang, Zhanjiang, Guangdong, China 524023; ^3^The First Clinical College, Guangdong Medical University, Zhanjiang, Guangdong, China 524023

## Abstract

Non-small-cell lung cancer (NSCLC) has a high incidence and mortality worldwide. Moreover, it needs more accurate means for predicting prognosis and treatments. Pyroptosis is a novel form of cell death about inflammation which was highly related to the occurrence and development of tumors. Despite having some studies about pyroptosis-related genes (PRGs) and cancer, the correlation has not been explored enough between PRGs and immune in NSCLC. In this study, we constructed a PRG model by WGCNA to access the prognosis value PRGs have. The testing cohort (*n* = 464) with four datasets from the GEO database conducted a survival analysis to confirm the stability of the prognostic model. The risk score and age are examined as independent prognostic factors. Based on the PRGs, we found multiple pathways enriched in immune in NSCLC. Separating samples into three subtypes by consensus cluster analysis, Cluster 3 was identified as immune-inflamed phenotype with an optimistic prognostic outcome. A three-gene PRG signature (BNIP3, CASP9, and CAPN1) was identified, and BNIP3 was identified as the core gene. Knockdown of BNIP3 significantly inhibited the growth of H358 cells and induced pyroptosis. In conclusion, the model construction based on PRGs provides novel insights into the prediction of NSCLC prognosis, and BNIP3 can serve as a diagnostic biomarker for NSCLC.

## 1. Introduction

Lung cancer is a malignant disease with high mortality and is still the main reason for cancer death worldwide [1]. And non-small-cell lung cancer (NSCLC) accounts for approximately 85% of gross lung cancer [2] which is the most common and serious subtype of malignant tumor in lung tissue. In histology, NSCLC mainly includes lung adenocarcinoma (65%) and lung squamous cell carcinoma (30%) [2]. Despite there being clinical advances that have been gained in molecular diagnosis, chemotherapy, and biologically targeted therapy being beneficial for overall survival in 5 years to NSCLC patients, the prognosis is still gloomy [3–5]. Standard lobectomy and sublobar resection are the common treatments for non-small-cell lung cancer patients in the early stage with about 60% 5-year survival rate [6], whereas the survival rate in 5 years of the NSCLC patients with clinical tumor-node-metastasis (TNM) stage IIIB or IV are only 7% and 2%, respectively [7]. Due to the heterogeneity of tumors and the complicated etiology mechanism of cancer, limited survival advances eagerly suggested more biologically prognostic targets have to be applied to risk stratification and treatment optimization for early-stage patients on clinic. Many studies have proved that mRNA can be a kind of signature to predict the prognosis of cancer precisely [8–10]. Through microarray gene expression profile analyses and screening, establishing prognostic gene signature may remedy the patient stratification of the present staging system and provide more personalized treatment.

Pyroptosis belonging to programmed cell death is related to inflammation [11], which is mediated by gasdermin (GSDM) under different stimuli [12] that plasma membrane would rupture rapidly and releases proinflammatory content from cells. The typical way to induce pyroptosis is to activate the inflammasomes [13] and mainly executed by the gasdermin family [14]. In addition, gasdermin E (GSDME) cleaved by caspase-3 can also induce pyroptosis [15, 16]. Generally, inflammatory vesicles, gasdermin proteins, and inflammatory cytokines are the key elements of pyroptosis that are related to the occurrence, development, invasion, and metastasis of tumors [17]. Nevertheless, the correlation between pyroptosis and cancer prognosis is not specific because of the dual effects of pyroptosis in tumor development. On the one hand, pyroptosis can suppress tumorigenesis and development through releasing inflammatory factors; on the other hand, pyroptosis creates a microenvironment for tumors with nutrition and accelerates the growth of tumors in various cancers [18–21]. In recent years, the dual effects of pyroptosis in cancer get popular and attract people to the study of the immune system [21]. And some studies indicated that pyroptosis would relate to the regulation of the tumor immune microenvironment [22].

The immune system plays a vital role in the NSCLC progression, and its components have been proved to be the major determinants of tumorigenesis and progression [23, 24]. The immune tumor microenvironment including various immune cells and stromal cells is regarded as a mark and a necessary part of NSCLC. And all the cells are related to immune-related genes (IRGs) and some immunomodulators that influence the prediction of cancer prognosis significantly [25–27]. Some immunotherapies have been applied to fight against tumors through the immune system [28, 29], but the valid part depends on the heterogeneity of the tumor microenvironment [30, 31]. The characteristic of NSCLC is a few mutations in the immune system which may be beneficial for the patients from immunotherapy [32]. Besides, it infers that the immune can stimulate the mechanism to work because of the better prognosis with the presence of inflammatory cells in NSCLC and other solid tumors [33, 34]. In the meanwhile, the stromal constituent in pulmonary tissue might play a role as a barrier in tumorigenesis by limiting the proliferation of tumor cells [35].

The classic immune subtype includes immune-inflamed, immune-excluded, and immune-desert phenotypes, which is related to response to anti-PD-1 and anti-PD-L1 antibodies [36, 37]. NSCLC mainly belongs to the immune-inflamed and immune-excluded phenotypes with a high tumor mutation burden (TMB) [38]. Besides, there is also another kind of LUAD molecular phenotype that displayed a difference in the tumor immune landscape, which contains the terminal respiratory unit, proximal proliferative, and proximal inflammatory subtypes [39], whereas the impacts of molecular subtypes on non-small-cell lung cancer and the clinical outcomes remain unanswered.

In this research, we acquired the mRNA expression profile of lung adenocarcinoma and lung squamous cell carcinoma with clinical information from the TCGA database. Then, we constructed a nomogram model with three prognostic pyroptosis-related genes and tested it in the validation cohort from the GEO database. Besides, we explored the correlation between risk scores and immune subtypes acquired from consensus cluster analysis.

## 2. Materials and Methods

### 2.1. Data Acquisition and Preprocessing

The RNA-seq matrix (count and FPKM value) in the TCGA-LUAD and TCGA-LUSC projects and corresponding clinical data was obtained from the Cancer Genome Atlas (TCGA) as the training cohort. And the validation dataset including GSE102287, GSE29013, GSE37745, and GSE50081 with its patient data was gained from the Gene Expression Omnibus (GEO) database. Additionally, the 146 pyroptosis-related genes and 2013 immune-related genes are from the GeneCards website and ImmPort portal, respectively. To ensure the consistency of expression level in each dataset, we removed the batch effect for all datasets by using the “sva” R package.

### 2.2. Weighted Gene Coexpression Network Analysis (WGCNA)

As a bioinformatics method, WGCNA can construct a gene coexpression network by utilizing the “WGCNA” package [40] in R software (version 4.0.5). Two datasets from the TCGA database were merged and converted into the form of the TPM value. Constructing a sample tree to exclude the outliers in the samples of the training cohort is beneficial for data stabilization. We chose the power of 4 as the soft threshold when the scale-free *R*^2^ is 0.9. And then, the adjacency matrix was defined by converting the expression profile based on soft-threshold. According to average linkage hierarchical clustering, TOM acquired from the adjacency matrix classified the modules with co-expression mRNAs. Next, we computed the module eigengene (ME) dissimilarity based on the ME expression profile after the Pearson correlation analysis. To merge the similar modules, we constructed ME tree after reclustering mRNAs in all modules while defining 0.6 as the height of the tree of merged modules.

In addition, we still need to screen out the module which highly associated with patient overall survival in these joined modules. Two analyses were conducted that one surveyed the correlation between the completed expression profile and ME expression level, and another explored the Pearson correlation between survival time which represented the clinical trait and mRNA expression profile (TPM value). Then, we explored the relationship between the results from two analyses in the module we selected to validate the significance of the module. To get the preliminary genes related to pyroptosis, we intersected the module genes and pyroptosis-related genes and then painted PPI with the intersecting genes by using Cytoscape software (version 3.8.2).

### 2.3. Screening of Prognostic Genes and Calculating Risk Value

The univariate Cox regression analysis was applied to access the prognostic significance of the pyroptosis-related genes and preserve them whose *p* value is lower than 0.05 by using the “survival” R package. Using LASSO analysis to calculate the coefficient of significant genes in each sample based on the “glmnet” R package. And cross-validation could prevent overfitting from happening to LASSO analysis. The computing formula of the LASSO approach is score = sum (each gene′s expression level × *λ*), and the prognostic genes signature were established based on it. According to the median risk score, the samples from the TCGA database were divided into high- and low-risk groups, also for the samples in the testing cohort. Afterward, the Kaplan–Meier analysis was executed to compare the OS in different risk groups in two datasets by using “survival” and “survminer” R packages. And “timeROC” R package painted ROC curve to predict the prognosis in 1, 3, and 5 years in the training dataset and 3, 6, and 9 years in the validation cohort.

### 2.4. Prognostic Module Construction and Enrichment Analysis

The multivariate Cox regression analysis about clinical characteristics is aimed at finding out the independent prognostic factors judging by *p* value (*p* value < 0.05 means significant). And we can obtain the range of hazard ratio, 95% confidence intervals (CIs), and *p* value of every clinical parameter via the “survival” R package. Then, the nomogram was constructed with the independent prognostic factors and visualized by using the “regplot” R package. The proportional hazard assumption was executed using Schoenfeld residual. We first divided the data into three layers based on the survival time of patients. Then, the Schoenfeld residual was calculated by “survival” R package. The calibration curves of 1, 5, and 8 years were validation and prediction of overall survival which were painted after multivariate Cox analyses and random sampling. And the samples fell into three groups with 230 samples randomly in each group. Gene set enrichment analysis (GSEA) was plotted by “ReactomePA” [41] and “clusterProfiler” [42] packages in R software. And we applied GSEA based on the genes in the “MEdarkgreen” module, pyroptosis, the intersection of the module, and pyroptosis-related and testing cohort.

### 2.5. Identification of Immune Subtype

To identify the immune subtype of the training cohort, the study used consensus clustering analysis and a series of related analyses. Applying the hierarchical cluster algorithm with 50 iterations to estimate the cluster robustness by the “ConsensusClusterPlus” R package [43]. The cumulative distribution function (CDF) curve calculated the increasing proportion area under it for determining the best cluster number. “survival” and “survminer” R packages for survival analysis were used to check the overall survival of clusters 1 and 2 with risk subgroup, respectively. In addition, the study ordered the significantly enriched pathways of each immune subgroup through KEGG functional analysis, and the R packages of “clusterProfiler,” “org.Hs.eg.db,” and “enrichplot” involved this analysis and plotting.

### 2.6. Immune Infiltration

ESTIMATE algorithm was conducted on R software to calculate the immune score, stromal score, and ESTIMATE score of overall patients with immune subgroups based on the counts value of the training dataset. In the meanwhile, identifying the cell type of each sample in the training cohort (TPM value) by CIBERSORT analysis is aimed at assessing the proportion of 21 immune cells in every patient according to the abundance of immune and stromal cells. And just kept the samples that the *p* value is under 0.05. Besides, single-sample GSEA (ssGSEA) was conducted to assess and compare the immune cell scores and abundance of immune functions in two risk groups, respectively. TIMER2.0 website was an online public database of immune, and we downloaded the plot pictures of the correlation between the expression level of prognostic genes and six immune cells. The immunohistochemistry images of three prognostic genes were obtained from the HPA website (The Human Protein Atlas). Eventually, we calculated the Pearson coefficient between immune checkpoints and prognostic PRGs.

### 2.7. The Screening of BNIP3

Rank forest (RF) algorithm was conducted in R software (version 4.0.5) by “randomForest” R package. And we deleted the genes whose importance score was less than 2. Separating samples from the TCGA database by “stringr” R package into the normal and tumor groups, we calculated the importance (mean decrease Gini) of each gene which was obtained after univariate Cox regression analysis. The genes were ranked in descending order by importance. The PPI network for searching hub genes was plotted by the “cytoHubba” application in Cytoscape software (version 3.8.2). The nodes with high score “cytoHubba” application calculated were red, and the nodes with low score were yellow. The ROC curves were obtained and visualized by “pROC” and “ggplot2” R packages in R software (version 4.0.5).

### 2.8. Cell Culture and Transfection

A lung cancer cell H838 was purchased from American Type Culture Collection (ATCC) and maintained in 1640 medium containing 10% fetal bovine serum (Gibco, Gland Island, USA) and 1% penicillin-streptomycin (Gibco, Gland Island, USA) and cultured at 37°C with 5% CO_2_. BNIP3-siRNA from Sangon Biotech (Shanghai, China) was used to silence the expression of BNIP3. In this study, the sequence of BNIP3-siRNA is sense: 5′- GAUUACUUCUGAGCUUGCATT -3′. H358 cells were seeded in 12-well plates at a density of 5 × 10^4^ cells/well and transfected 24 hours later. BNIP3-siRNA or NC-siRNA was transfected to a final concentration of 20 nM using siRNA transfection reagent (Polyplus, France). Finally, the transfected cells were detected after 48 hours.

### 2.9. Cell Apoptosis Assay

H358 cells were treated with transfection reagents BNIP3-siRNA and NC-siRNA for 48 hours. After 48 hours, cells were collected and then incubated for 30 min at room temperature in the dark using the FITC-labeled membrane-linked protein-V and PI (BD Biosciences) in the apoptosis kit. Eventually, apoptosis was detected using flow cytometry.

### 2.10. Western Blot

Proteins were extracted from cells and cell lysates were prepared using RIPA lysate with PMSF (Solarbio, Beijing, China), and then, protein quantification was performed using a BCA protein assay kit (Sangon Biotech, Shanghai, China). Proteins were then separated by 12% SDS-PAGE and transferred to nitrocellulose membranes. The membranes were blocked with 5% bovine serum albumin (BSA) for 1 h and then incubated with primary antibody GAPDH, BNIP3, GSDMD, and caspase-8 overnight at 4°C. The next day, after washing the membrane three times with TBST, the membrane was incubated for 1 h at room temperature with horseradish peroxidase-labelled secondary antibody (1 : 4000), followed by three washes with TBST. Finally, the results of western blot were detected by BeyoECL Moon (Beyotime Biotechnology, Shanghai, China).

### 2.11. Immunohistochemistry (IHC)

Tumor tissues were fixed with 4% paraformaldehyde, embedded in paraffin, and prepared into 5 *μ*m thick sections. The sections were subsequently dewaxed with xylene and dehydrated in a gradient concentration of alcohol solution. For immunohistochemical experiments, antigen retrieval was performed with 0.01 M sodium citrate (pH: 6.0), and endogenous peroxidase was blocked by adding 0.3% hydrogen peroxide (H_2_O_2_) and incubating in 10% goat serum albumin for 30 min. Sections were then incubated with primary antibody (BNIP3) at 4°C overnight. The next day, after incubation with HRP-conjugated anti-rabbit secondary antibody for 1 h, samples were incubated with 3,3′-diaminobenzidine (DAB), sections were counterstained with Mayer hematoxylin, dehydrated, cleared with xylene, and finally blocked with neutral resin and then observed on a multifunctional microtome (BIOTEK).

### 2.12. Statistical Analysis

All bioinformatics statistical analyses in the study were conducted using R software (version 4.0.5). The survival analysis was performed with a Kaplan-Meier assay and a log-rank test. To test the nomogram, we conducted proportional hazard assumption by calculating the Schoenfeld residual. The Mann–Whitney test was used for the validation of ssGSEA comparing. All experiments were repeated at least three times and the data were expressed as mean ± SEM. ANOVA or *t*-test was used to determine the statistical significance of the control and experimental groups. In the investigation, data were considered statistically significant when *p* < 0.05.

## 3. Results

### 3.1. Construction of Weighted Coexpression Network and Identification of Trait-Module

**Figure 1:**
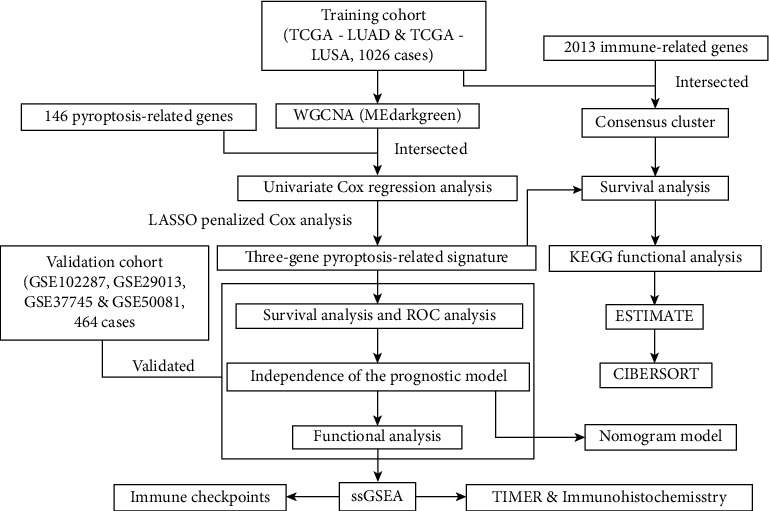
The flow chart of the overall study.

**Figure 2:**
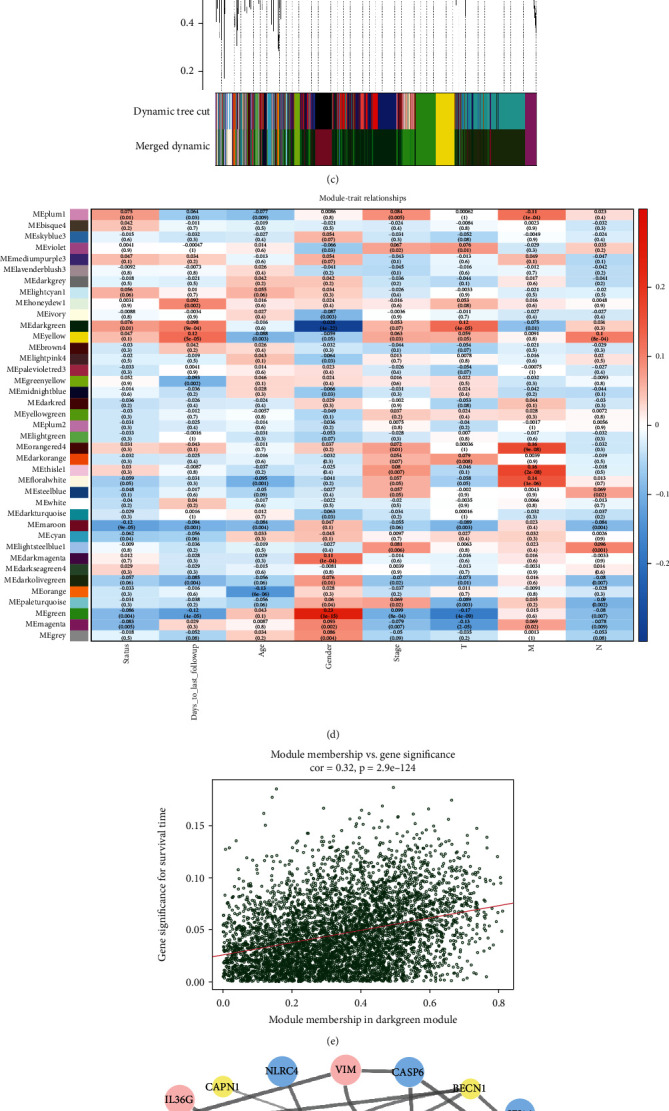
Coexpression network construction and identification of module related with clinical traits in non-small-cell lung cancer (NSCLC) patients. (a) Analysis of the scale-free fit index for different soft-thresholding powers. (b) Analysis of the correlation between mean connectivity and various soft-thresholding powers. (c) Dendrogram of the genes after merging modules and reclustering. Every color represents a module. (d) The relationships between modules and clinical characteristics of NSCLC; red means positive correlation, and blue means negative correlation. (e) Analysis of gene significance for module membership in the dark green module. The red line is a fitted curve. (f) The PPI network of pyroptosis-related genes in the “MEdarkgreen” module. The color and size of genes represent the degree value (database annotated). The big blue dots represent genes with a high degree, the small yellow dots are the genes with a low degree, and pink dots are the genes without a degree. The grey lines links dots represent the combined score and the thicker with higher relevance.

The flow chart of the overall design in this study was shown in Figure 1. In this study, mRNA expression profiles with 1145 cases and 1026 pieces of patient clinical information from the TCGA-LUAD and TCGA-LUSC projects were assembled as the training cohort, and 4 datasets from the GEO database containing 464 samples were selected as our validation cohort after removing batch effect. Conducting WGCNA to construct a weighted coexpression network, the genes which have similar expression tendencies would be divided into one module. Every module was named with corresponding colors. When the scale-free topology fit index *R*^2^ = 0.9, the power of soft-thresholding value reached 4. Therefore, we calculated the adjacency matrix and constructed the co-expression network based on the power value of soft-thresholding (Figures 2(a) and 2(b)). To decrease the part of a subdivision in the network, we computed the module eigengenes (MEs) and the number of modules decreased from 59 to 39 (Figure 2(c)). The relation of modules with clinical traits was presented in the form of *p* value in the heat map (Figure 2(d)). Due to the positive correlation between module and survival time, we selected the “MEdarkgreen” module as our research object. To certify the reliability of the “MEdarkgreen” module, we analyzed the Pearson correlation between gene significance of the selected module and module membership (Figure 2(e)). Afterward, the genes in the “MEdarkgreen” module were intersected with pyroptosis-related genes and 35 pyroptosis-related genes in the module were obtained. Eventually, the interaction of these genes was analyzed by the STRING website (https://www.string-db.org/) and visualized the protein-protein interaction (PPI) network with Cytoscape software (version 3.8.2) (Figure 2(f)).

### 3.2. Prognostic Gene Identification and Nomogram Model Establishment

**Figure 3:**
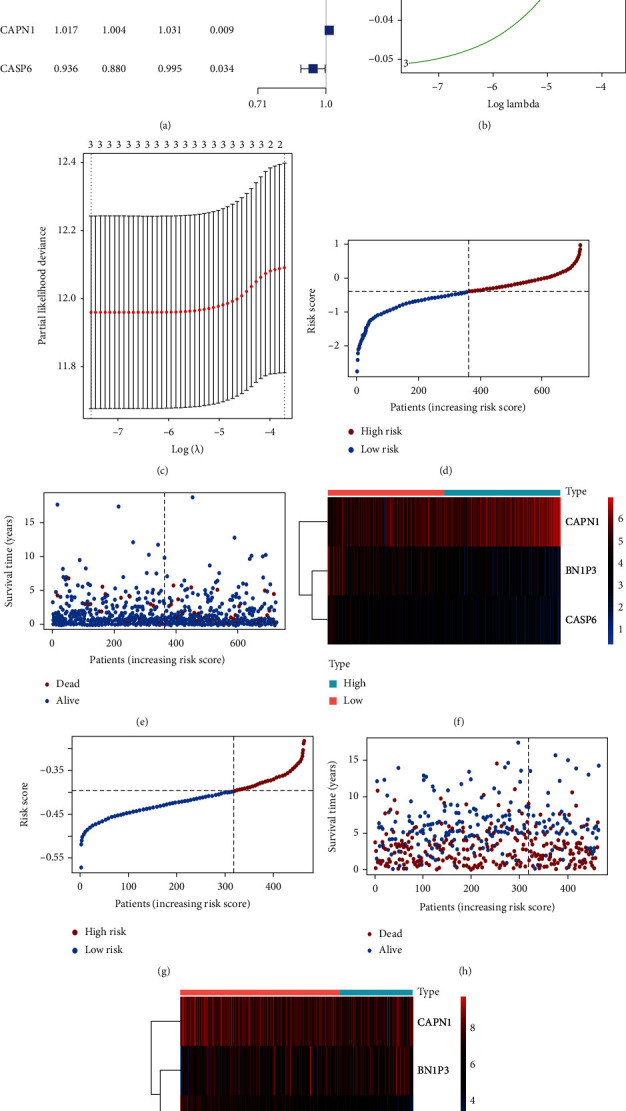
The prognostic genes identification and the correlation between risk score and survival condition with validation. (a) The forest plot of prognostic genes is based on the univariate Cox regression analysis. HR: hazard ratio; HR.95L, HR.95H: hazard ratio 95% confidence interval. (b) Distribution of LASSO coefficients of the three prognostic genes in the training cohort. (c) Selection of the best parameter (lambda) in the lambda sequence. (d) The distribution of risk groups based on risk score in samples in the training cohort. (e) The survival status of NSCLC patients in the training cohort in different risk groups. (f) The expression level of prognostic genes in the high- and low-risk groups in the training cohort. (g–i) Validation for aforementioned analyses about risk score based on GEO dataset.

**Figure 4:**
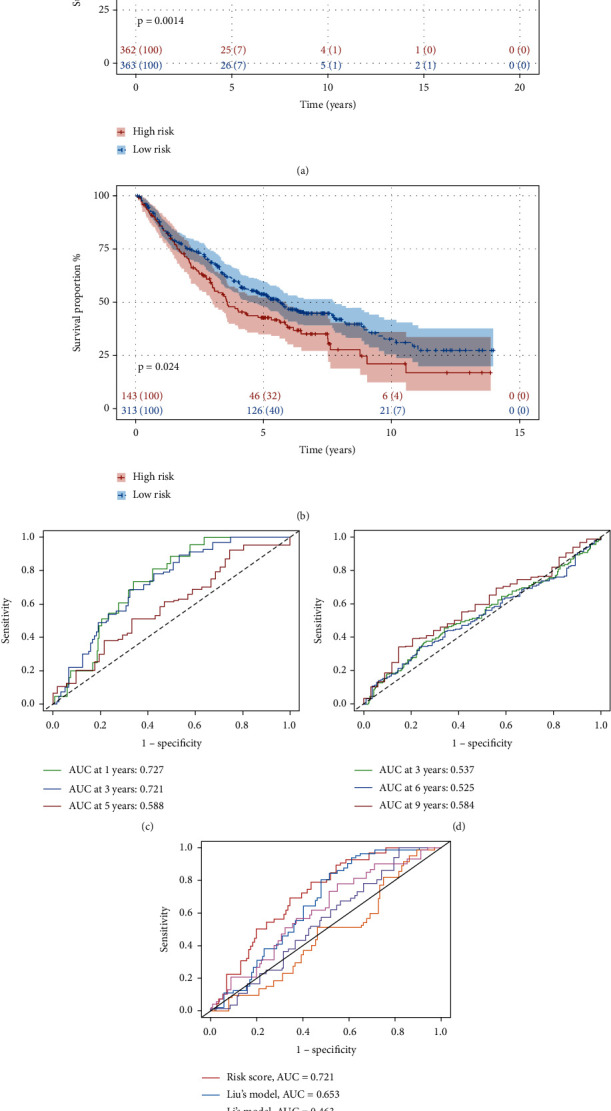
Survival analysis for training cohort and testing cohort. (a) Kaplan-Meier survival analysis of samples in different risk groups in the training cohort. (b) ROC curve for 1, 3, and 5 years of survival time prediction. (c, d) The testing of survival analysis in validation dataset from GEO database. (e) ROC curve for comparing the prognostic model with other published models.

Applying univariate Cox regression analysis to 35 pyroptosis-related genes to find out the prognosis-related genes (Table S1), there were three genes including CAPN1, BNIP3, and CASP6 significantly related with prognosis (*p* value < 0.05) after analysis (Figure 3(a)). To further confirm the relation of three genes with overall survival (OS) in NSCLC patients, the LASSO regression model was constructed and cross-validation prevented the overfitting from LASSO analysis (Figures 3(b) and 3(c)). The risk coefficient of each sample was calculated after LASSO regression analysis, and the sample would divide into the high- and low-risk groups according to the median of the risk score (Figure 3(d)). The next step was to explore the correlation between risk score and survival status. As shown in Figure 3(e), the higher risk score is accompanied by more dead patients. Besides, we also checked the expression level of three prognostic genes in two risk groups, and only CAPN1 was expressed obviously in the high-risk group (Figure 3(f)). The detailed survival condition in two risk groups was presented in Figure 4(a), in which we can observe the relationship among survival time, survival status, and risk groups through survival analysis. The two groups had a significant difference in survival condition (*p* value < 0.05), and patients in the low-risk group got a better prognosis. The ROC curves with 1, 3, and 5 years were painted to validate the accuracy of predicting prognosis, and the more area under the curve (AUC) means the higher reliability for prediction (Figure 4(b)). For improving the accuracy of the aforementioned analyses about survival conditions, we used the testing cohort which had merged four datasets from the GEO database to validate. The results were consistent with the training cohort except ROC was poor on reliability (Figures 3(g)–3(i), 4(c), and 4(d); Table S2). Afterwards, we compared our prognostic model with some published models by painting ROC curve (Figure 4(e)). The AUC value of our prognostic model was the highest in all of the compared models in 3 years (AUC = 0.721).

**Figure 5:**
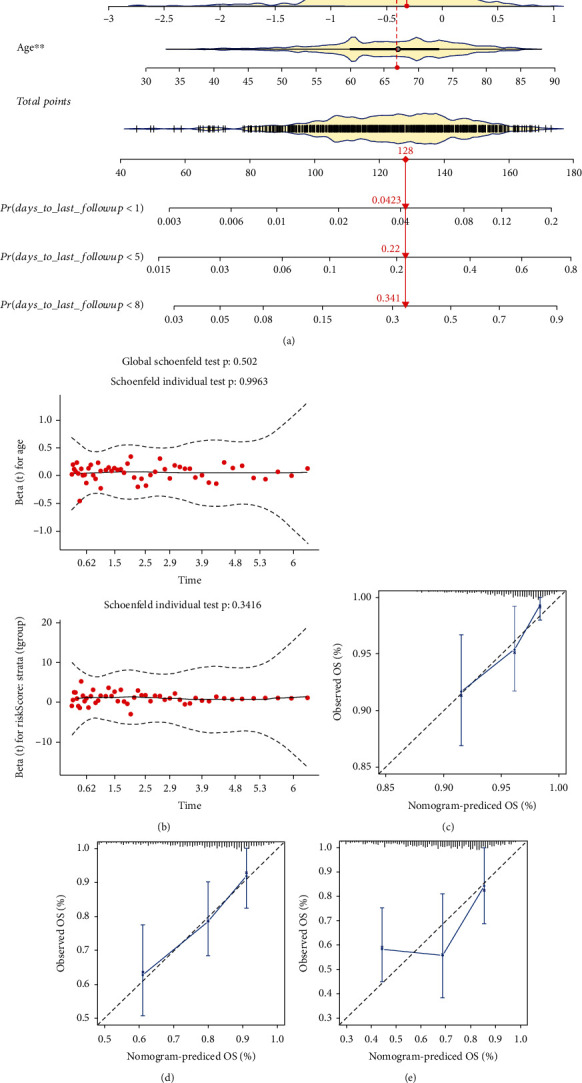
Construction of nomogram module and calibration. (a) Nomogram construction with independent prognostic factors (^∗^*p* value < 0.05; ^∗∗^*p* value < 0.01). (b) The Schoenfeld residuals of two clinical factors (age and risk scores) for proportional hazard assumption to test the nomogram. (c–e) The calibration curve for the nomogram model with 1, 5, and 8 years, respectively. OS: overall survival.

To construct the nomogram model, we firstly processed independence prognosis analysis by using the method of multivariate Cox regression analysis to screen the clinical parameters, age and risk score were the two clinical characteristics whose *p* value was under 0.05 (Table 1). Then, the nomogram model was built based on two independent prognostic factors to predict the survival rate in 1, 5, and 8 years (Figure 5(a)). The proportional hazard assumption was conducted to test the nomogram by calculating the Schoenfeld residual (Figure 5(b)). The *p* values of the two independent prognostic parameters are higher than 0.05, which means the proportional hazard assumption is effective and the establishment of nomogram is a success. The calibration curves of 1, 5, and 8 years presented the correlation between the rate of nomogram-predicted OS and observed OS and confirmed the reliability of prediction power in the nomogram model (Figures 5(c)–5(e)).

### 3.3. Gene Set Enrichment Analysis

**Figure 6:**
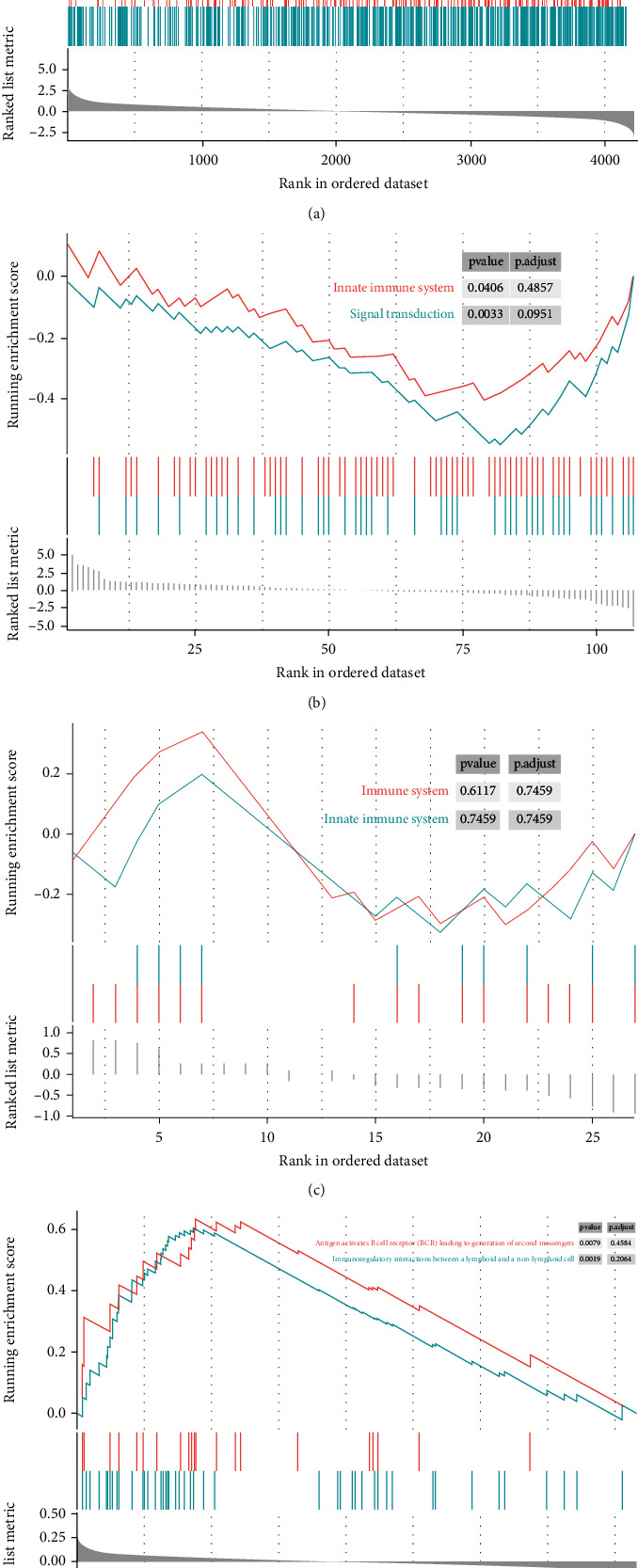
Gene set enrichment analysis. (a) Gene set enrichment analysis (GSEA) for “MEdarkgreen” module genes. (b) GSEA for all pyroptosis-related genes based on training cohort. (c) GSEA for pyroptosis-related genes in the “MEdarkgreen” module. (d) GSEA for genes after intersecting the “MEdarkgreen” module and validation cohort.

Processing gene set enrichment analysis to four datasets mentioned in the materials and methods part to search the pathways and screen by calculating the *p* value (*p* value < 0.05). As observed, the pathways of the “Immune system” and “Innate immune system” appeared multiple times in three datasets (Figures 6(a)–6(c)). We found that “Immunoregulatory interactions between a lymphoid and a nonlymphoid cell” and “PD-1 signaling” pathways are significantly enriched in the validation cohort (Figure 6(d)). Therefore, we deduced that pyroptosis may have a correlation with immune and explored more about immune by analyzing the pyroptosis-related expression profile.

### 3.4. Survival Analysis and KEGG Pathway Enrichment Analysis with Immune Subgroups

**Figure 7:**
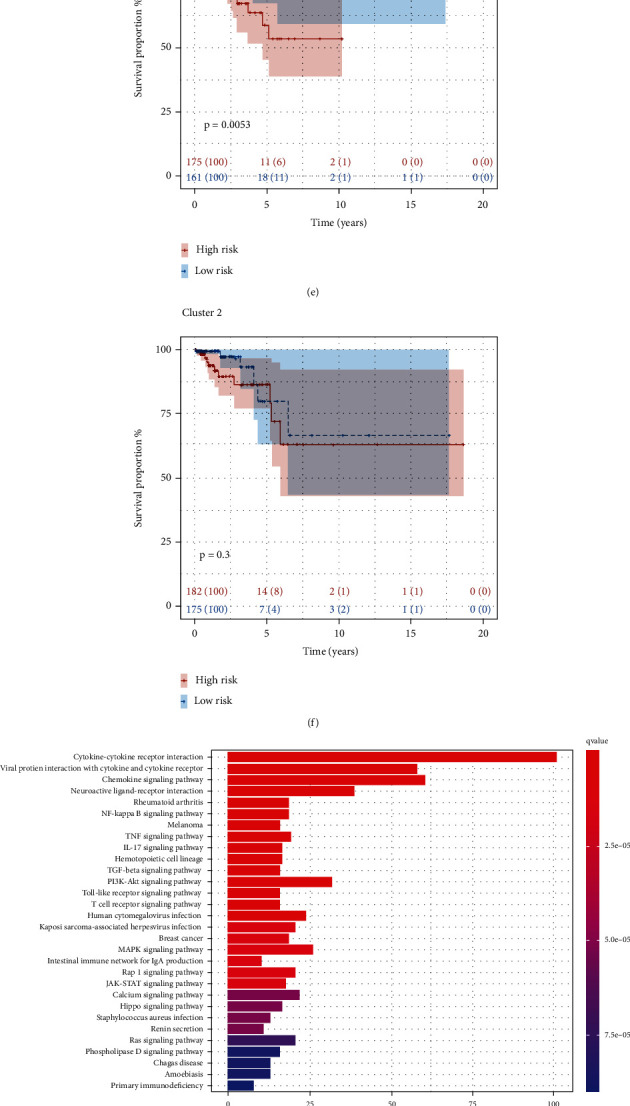
Identification of immune subtype and KEGG (Kyoto Encyclopedia of Genes and Genomes) function enrichment analysis. (a) The relationship between CDF (cumulative distribution function) area and consensus index. (b) The relative change in area under CDF curve for *k*-means. (c) The survey of consensus matrix when *k* = 3 and sample distribution. (d) Survival analysis of immune subtype in the training cohort. C1: cluster 1; C2: cluster 2; C3: cluster 3. (e, f) Kaplan-Meier survival analysis of cluster 1 and cluster 2. (g–i) KEGG functional enrichment analysis for three clusters, respectively.

Firstly, the list of the immune-related genes was obtained from the ImmPort website and intersected with the expression profile of the training cohort. And consensus clustering analysis identified the immune-subtype based on the immune-related expression matrix. As shown in Figure 7(a), the area under CDF curves no longer increased rapidly after *k* = 3. And the same result can be deduced by analyzing the relative change in area under the CDF curve (Figure 7(b)). Based on the matrix of clustering heat map, we decided to divide the patients into three subgroups (Figure 7(c)). To search the subgroups correlation with overall survival, we conducted survival analysis to the complete expression profile grouped by consensus analysis and the difference between the three subgroups was significant (*p* value = 0.032) (Figure 7(d)). For deep exploring the influence of risk score in clustering subgroups, we applied survival analysis to the three subgroups, respectively (Figures 7(e) and 7(f)). As observed in the figures, the prognosis in the low-risk group was better than in the high-risk group in cluster 1 obviously (*p* = 0.0053), but insignificantly in cluster 2 (*p* value = 0.3). Besides, KEGG pathway enrichment analysis was used for the three subgroups, and some enriched pathways were left (*p* value < 0.05) (Figures 7(g)–7(i)). In cluster 1, the pathway is mainly enriched in “Cytokine-cytokine receptor interaction,” “Viral protein interaction with cytokine and cytokine receptor,” “Chemokine signaling pathway,” and so on, also in cluster 2 but a little different in cluster 3. The pathways including “Renin secretion,” “Cytokine-cytokine receptor interaction,” and “Renin-angiotensin system” were enriched in cluster 3.

### 3.5. Immune Infiltration

**Figure 8:**
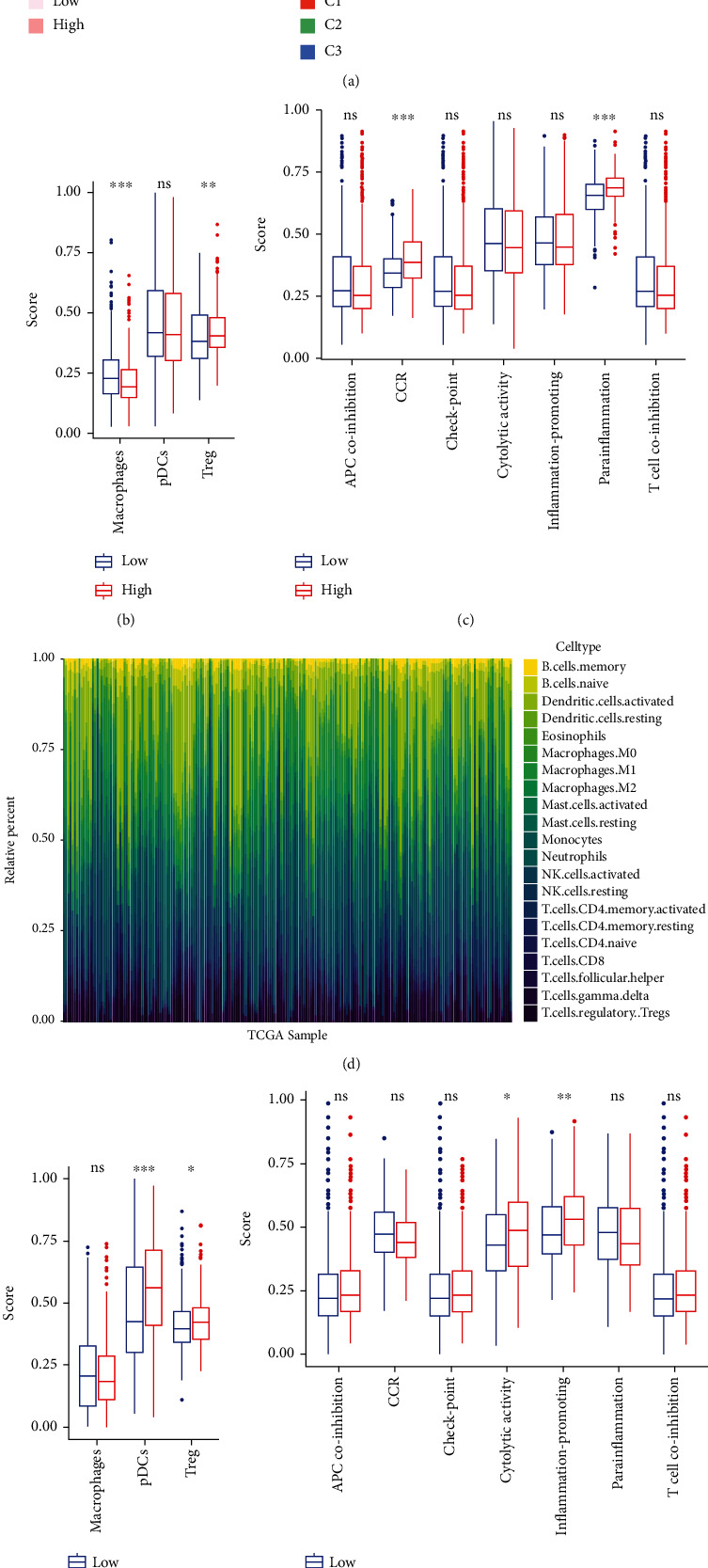
Immune infiltration analysis. (a) ESTIMATE analysis of training cohort with risk and immune subgroups. C1: cluster 1; C2: cluster 2; C3: cluster 3. (b, c) The enrichment situation of immune cells in training cohort (b) and testing cohort (c) by ssGSEA (ns: not significant; ^∗^*p* value < 0.05; ^∗∗^*p* value < 0.01; and ^∗∗∗^*p* value < 0.001). (d) CIBERSORT analysis of entire training cohort. (e, f) The enrichment analysis result of immune functions in training cohort (e) and validation dataset (f) after ssGSEA (ns: not significant; ^∗^*p* value < 0.05; ^∗∗^*p* value < 0.01; and ^∗∗∗^*p* value < 0.001).

Using ESTIMATE algorithm to calculate the scores of immune, stromal, and estimate and visualized on heat map image with immune cluster (Figure 8(a)). And both the immune score and stromal score in cluster 2 were the lowest, and it explained that both the immune and stromal cells are low-content in the samples of cluster 2. Thus, we deduced that cluster 2 was regarded as the immune-desert phenotype. The immune score was assessed highly in clusters 1 and 3. Nevertheless, the estimate score in cluster 1 was low which means that the immune and stromal scores are not enriched. The estimate score in cluster 3 was more stable and higher than in cluster 1. Therefore, we supposed that cluster 3 is the immune-inflamed phenotype. Afterward, the CIBERSORT analysis was conducted to check the proportion of immune cells in 21 types in each case and screen the samples whose *p* value was higher than 0.05 (Figure 8(d)). To deeply discuss the relation of immune status with risk scores, the ssGSEA analysis was performed on training (Figures 8(b) and 8(c)) and testing cohort (Figures 8(e) and 8(f)). As observed in the training cohort, the enrichment scores of macrophages and Treg were significantly different in the high- and low-risk groups (*p* value < 0.01) and the score of immune function including CCR and Para inflammation had an obvious difference (*p* value < 0.001). The enrichment scores of pDCs, Treg, cytolytic activity, and inflammation-promoting in the validation dataset were significantly different (*p* value < 0.05). In addition, we downloaded some images about immune cells and immunohistochemistry from the TIMER2.0 website and the HPA database (The Human Protein Atlas), respectively. As shown in Figure S1, we can observe the correlation between six immune cells and three prognostic genes in lung adenocarcinoma and lung squamous carcinoma, but most of them had a slightly negative correlation. Figure S2 showed the images of immunohistochemistry of three prognostic genes in normal tissues, lung adenocarcinoma tissues, and lung squamous carcinoma tissues, respectively. And there were obvious differences between normal tissues and tumor tissues. Eventually, we explored the relevance between PRGs and immune checkpoints. The condition of the expression level of 35 PRGs (before univariate Cox analysis) in immune subtypes was presented in Figure S3A. The BNIP3 was expressed significantly in clusters 1 and 3 but had a low expression in cluster 2. The CAPN1 was expressed highly in cluster 1 but expressed low in cluster 3. And the CASP6 was expressed highly in cluster 3. The expression level of TIGIT and LAG3 in the immune checkpoints in the two risk groups is different significant, and both of them were expressed higher in the high-risk group (Figure S3F). Therefore, we checked the Pearson correlation coefficients between the two immune checkpoints and prognostic PRGs (Figure S3B-E, G, H). The BNIP3 had an obvious negative correlation with the two immune checkpoints, respectively. The CAPN1 only negatively correlated with TIGIT, whereas the CASP6 did not present any relation with the two immune checkpoints.

### 3.6. BNIP3 Is Highly Expressed in Lung Adenocarcinoma, and Knockdown of BNIP3 Induces Lung Cancer Cell Pyroptosis

Eventually, we screened the genes further based on the prognostic model. The three prognostic signatures' expression levels were up-regulated in the tumor group (Figure S4A). The random forest (RF) algorithm was conducted to calculate the importance of each gene after the univariate Cox regression analysis (Figure S4C). The importance of genes means the contribution degree of genes to the prognostic model. And BNIP3 contributes most in the three prognostic genes (mean decrease Gini = 9.78), CAPN1 contributes least in the three prognostic genes (mean decrease Gini = 2.29). Subsequently, the PPI network was constructed to search the hub genes (Figure S4B). However, only BNIP3 and CASP6 were selected in the prognostic genes to build the hub genes network. Hence, we removed CAPN1 and continued to explore the details of BNIP3 and CASP6. Plotting ROC curves based on the expression level of BNIP3 and CASP6, respectively (Figure S4D, E), the AUC value of BNIP3 (AUC = 0.853) was higher than the value of CASP6 (AUC = 0.848). In summary, BNIP3 became our final target signature to explore.

**Figure 9:**
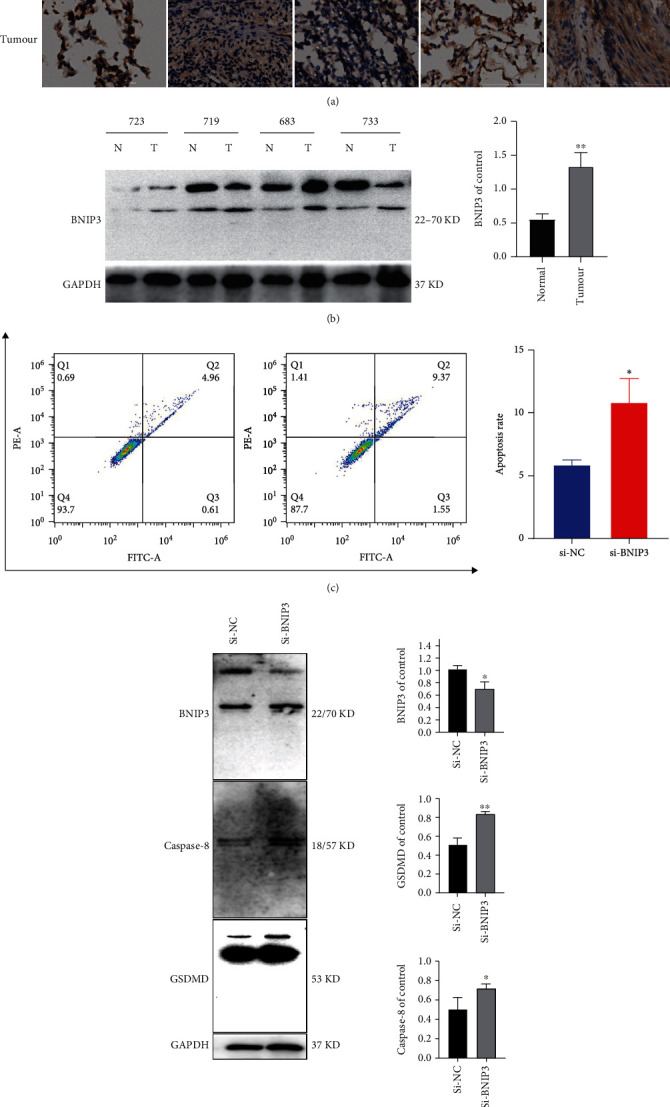
Knockdown of BNIP3 induces pyroptosis in lung cancer cells. (A) 656, 683, 703, 714, and 715 are patient numbers, immunohistochemical assay to compare BNIP3 expression in normal lung tissue and lung cancer tissue. (b) The expression of normal lung tissue protein and lung cancer tissue protein expression were detected by western blotting assay. Densitometry of the ration of BNIP3 was shown as bar chart. All data were representative of at least three independent experiments and presented as mean ± SD, ^∗∗^*p* < 0.01 compared with the control. 723, 719, 683, and 733 are patient numbers. (c) Using flow cytometry to detect cell death of H358 after knockdown BNIP3. (d) Pyroptosis-related proteins were detected by western blotting assay. Densitometry of the ration of protein was shown as bar chart. All data were representative of at least three independent experiments and presented as mean ± SD, ^∗^*p* < 0.05, ^∗∗^*p* < 0.01 compared with the control.

To further explore the role of BNIP3 in lung cancer cells, five pairs of human lung cancer tissues and their paraneoplastic tissues were incubated with BNIP3 as primary antibody, and the results of immunohistochemistry showed that BNIP3 was highly expressed in lung adenocarcinoma compared with normal tissues (Figure 9(a)). We also took 4 pairs of human lung cancer tissues and their paraneoplastic tissues and extracted the proteins for western blot experiments, and the results showed that BNIP3 were upregulated in lung cancer tissue proteins (Figure 9(b)). After treating H358 cells with transfection reagent for 48 h, we examined the cell death using an apoptosis kit in order to detect the effect of knocking down BNIP3 on H358 cells, and the results showed that the cell death rate was significantly increased in the knockdown BNIP3 group (Figure 9(c)). Knocking down BNIP3 with transfection reagent, then we extracted proteins and detected GSDMD and caspase-8 by western blot, and the results showed that knocking down BNIP3 increased GSDMD and caspase-8, indicating that knocking down BNIP3 could induce pyroptosis in lung cancer cells H358 (Figure 9(d)).

## 4. Discussion

Pyroptosis induction can thoroughly remove neoplastic cells in multiple cancers [44]. However, due to the activation of pyroptosis, some inflammatory mediators will be released which might promote the occurrence and progression of cancer [45, 46]. The respiratory system is sensitive to pyroptosis induction [47]. The piperlongumine analogue L50377 induced pyroptosis by stimulating reactive oxygen species (ROS) to mediate the suppression of NF-*κ*B in NSCLC [48]. Additionally, the downregulation of lncRNA-XIST would activate pyroptosis mediated by the miR-335/SOD2/ROS signal pathway to suppress the development of NSCLC [49]. Many studies suggested that ROS is related to pyroptosis [50, 51]. Nevertheless, the mechanism of how pyroptosis influences NSCLC and what kind of regulations the predictors do via the pathways are not clear until now. Therefore, we constructed a prognostic model based on three pyroptosis-related genes by using bioinformatics methods which were validated in the testing cohort to prove its availability and benefit to early diagnosing for NSCLC patients.

In this investigation, we determined three pyroptosis-related genes including CAPN1, BNIP3, and CASP6 as prognostic signatures of NSCLC based on the expression profile with 146 pyroptosis-related genes from the TCGA database, which are overexpressed in the tumor tissues. Calpain 1 (CAPN1) is a type of cysteine activated by calcium with proinflammation [52], which is widely expressed in vivo and has been proved as a promoter of cancer progression that is significantly related to poor prognosis [53–55]. The Calpain family where CAPN1 belongs can influence the malignancy phenotype of lung cancer cells by degrading proteins according to some reports [56–59] and also is involved in various cellular processes containing cell signal transduction and apoptosis, etc. [52]. And several studies have verified the importance of the Calpain family in tumor migration and invasion [60]. Meanwhile, CAPN1 may be a biomarker of tumor or a potential target used to diagnose and treat lung adenocarcinoma [61]. The CAPN1 rs17583C>T was related to a better prognosis, which provided certification of the functional relationship between genetic mutation and the better clinical outcomes [62]. BNIP3, a member of the Bcl-2 protein family with mitochondrial BH3 [63], affects different ways of cell death in hypoxic conditions [64] and also various metabolic pathways [65, 66]. BNIP3 is regarded as a proapoptotic protein, whose dysregulated expression is related to mitophagy, autophagy, and pyroptosis [67–69]. Tumor cells are sensitive to cisplatin and gemcitabine when BNIP3 upregulates [70]. Interestingly, the expression level of BNIP3 will elevate in early-stage adenocarcinoma but decrease in metastasis progression. And the deletion of BNIP3 can increase angiogenesis which promotes tumorigenesis and the metastasis of breast cancer [71]. Also, BNIP3 was inferred as an independent prognostic factor that related to autophagy in early-stage NSCLC but not clear in advanced lung cancer [63]. CASP6 was suggested that may be related to apoptosis [72] which attends to the occurrence and development of cancer by promoting the activation of the ways of programmed cell death in tumor issues [73]. And it has been detected in some reports about pyroptosis as a prognostic biomarker in cancer [74, 75]. Besides, CASP6 can mediate the activation of the innate immune system and inflammasomes [76]; also, its mutation associated with tumors can decrease the overall catalytic turnover [77].

The result of GSEA based on the pyroptosis-related genes suggested that the immune may play a relevant role in pyroptosis and non-small-cell lung cancer and the pathways are mainly enriched in the immune system and innate immune system. The inflammation induced by pyroptosis can activate antitumor immunity and synergy with checkpoint blockade [78]. The inflammasome is the key mediator of lung immunity that would be regulated to release caspase-1 [79]. The activation of caspase-1 will trigger the pyroptosis [80]. Caspase-1-dependent process and secrete IL-1*β* and IL-18 by means of inflammatory caspases activation and the innate immune responses induced by macrophages [81, 82]. Besides, many studies identified that CD8+ T cells and NK cells can suppress tumors through the induction of pyroptosis of tumor cells in the immune microenvironment, and the tumor cells under pyroptosis would recruit immune cells for suppressing tumors, too [18, 78]. However, inducing pyroptosis cannot be beneficial for all immunotherapy modalities and sometimes would be required to work with ICIs to kill the cold tumor cells efficiently [78].

The immune molecular phenotype may help identify the patients who would benefit from immunotherapy [83]. In this research, we identified three immune subtypes by consensus cluster analysis. Cluster 2 was supposed to be the immune-desert phenotype because of the low immune and stromal scores, and cluster 3 was the immune-inflamed phenotype with the high immune score. BNIP3 had a low expression in the high-risk group and cluster 2. Meanwhile, the high-risk group and cluster 2 had poor prognoses compared with the low-risk group and cluster 3, respectively. Hence, we supposed that the low expression level of BNIP3 suggested the immune-desert phenotype and the high level of immune cell infiltration. And the high-risk group had a poor prognosis because of it.

In conclusion, we constructed a prognostic nomogram model with three pyroptosis-related genes and validated it in another dataset and also explored the correlation between pyroptosis-related and immune microenvironment based on immune subgroups at the same time. A three-gene PRG signature (BNIP3, CASP9, and CAPN1) was identified, and BNIP3 was identified as the core gene. Knockdown of BNIP3 significantly induced pyroptosis. In conclusion, the model construction based on PRGs provides novel insights into the prediction of NSCLC prognosis, and BNIP3 can serve as a diagnostic biomarker for NSCLC. However, there are still some limitations in this research. The patient data was all acquired from the public database so that we did not use more data to strengthen the reliability of the prognostic model. The lack of experiments *in vivo* also makes our study results unable to be further verified. Therefore, we need forward-looking studies to improve the availability of this prognostic model.

## Figures and Tables

**Table 1:** The multivariate Cox regression analysis of clinical parameters in the training cohort for independence prognosis analysis.

Hazard ratio				
Variable	HR	HR.95L	HR.95H	*p* value
Age	1.071	1.029	1.114	<0.001
Gender	0.986	0.541	1.795	0.963
Stage II	2.234	0.682	7.318	0.184
Stage III	2.167	0.304	15.443	0.440
T2	0.958	0.489	1.876	0.900
T3	0.528	0.117	2.375	0.405
T4	1.369	0.232	8.090	0.729
N1	0.629	0.190	2.083	0.448
N2	1.188	0.193	7.315	0.853
riskScore	2.552	1.376	4.734	0.003

Abbreviations: HR: hazard ratio; HR.95L, HR.95H: hazard ratio 95% confidence interval.

## Data Availability

The data used to support the findings of this study are included within the article.

## Supplementary Materials

Supplementary Materials Figure S1: immune infiltration supplements of prognostic genes. Figure S2: immunohistochemistry plots of prognostic genes from the HPA website. Figure S3: the relation between pyroptosis-related genes (PRGs) and immune. Table S1: univariate Cox regression analysis of PRGs in The Cancer Genome Atlas. Table S2: the survival situation in the risk groups in the training and testing cohorts.

## References

[1] Sung H., Ferlay J., Siegel R. L. (2021). Global cancer statistics 2020: GLOBOCAN estimates of incidence and mortality worldwide for 36 cancers in 185 countries. *CA: a Cancer Journal for Clinicians*.

[2] Chen W., Zheng R., Baade P. D. (2016). Cancer statistics in China, 2015. *CA: a Cancer Journal for Clinicians*.

[3] Shaipanich T., McWilliams A., Lam S. (2006). Early detection and chemoprevention of lung cancer. *Respirology*.

[4] Park K. S., Raffeld M., Moon Y. W. (2014). CRIPTO1 expression in EGFR-mutant NSCLC elicits intrinsic EGFR-inhibitor resistance. *The Journal of Clinical Investigation*.

[5] Smeltzer M. P., Wynes M. W., Lantuejoul S. (2020). The International Association for the Study of Lung Cancer global survey on molecular testing in lung cancer. *Journal of Thoracic Oncology*.

[6] Okami J., Ito Y., Higashiyama M. (2010). Sublobar resection provides an equivalent survival after lobectomy in elderly patients with early lung cancer. *The Annals of Thoracic Surgery*.

[7] Goldstraw P., Crowley J., Chansky K. (2007). The IASLC Lung Cancer Staging Project: proposals for the revision of the TNM stage groupings in the forthcoming (seventh) edition of the TNM classification of malignant tumours. *Journal of Thoracic Oncology*.

[8] Guo H., Cai J., Wang X. (2020). Prognostic values of a novel multi-mRNA signature for predicting relapse of cholangiocarcinoma. *International Journal of Biological Sciences*.

[9] Peng K., Chen E., Li W. (2020). A 16-mRNA signature optimizes recurrence-free survival prediction of stages II and III gastric cancer. *Journal of Cellular Physiology*.

[10] Zhu C. Q., Ding K., Strumpf D. (2010). Prognostic and predictive gene signature for adjuvant chemotherapy in resected non-small-cell lung cancer. *Journal of Clinical Oncology*.

[11] Kovacs S. B., Miao E. A. (2017). Gasdermins: effectors of pyroptosis. *Trends in Cell Biology*.

[12] Maltez V. I., Tubbs A. L., Cook K. D. (2015). Inflammasomes coordinate pyroptosis and natural killer cell cytotoxicity to clear infection by a ubiquitous environmental bacterium. *Immunity*.

[13] Xue Y., Enosi Tuipulotu D., Tan W. H., Kay C., Man S. M. (2019). Emerging activators and regulators of inflammasomes and pyroptosis. *Trends in Immunology*.

[14] Broz P., Pelegrin P., Shao F. (2020). The gasdermins, a protein family executing cell death and inflammation. *Nature Reviews. Immunology*.

[15] Wang Y., Gao W., Shi X. (2017). Chemotherapy drugs induce pyroptosis through caspase-3 cleavage of a gasdermin. *Nature*.

[16] Rogers C., Fernandes-Alnemri T., Mayes L., Alnemri D., Cingolani G., Alnemri E. S. (2017). Cleavage of DFNA5 by caspase-3 during apoptosis mediates progression to secondary necrotic/pyroptotic cell death. *Nature Communications*.

[17] Kolb R., Liu G. H., Janowski A. M., Sutterwala F. S., Zhang W. (2014). Inflammasomes in cancer: a double-edged sword. *Protein & Cell*.

[18] Zhang Z., Zhang Y., Xia S. (2020). Gasdermin E suppresses tumour growth by activating anti-tumour immunity. *Nature*.

[19] Cui J., Zhou Z., Yang H. (2019). MST1 suppresses pancreatic cancer progression via ROS-induced pyroptosis. *Molecular Cancer Research*.

[20] Wang L., Qin X., Liang J., Ge P. (2021). Induction of pyroptosis: a promising strategy for cancer treatment. *Frontiers in Oncology*.

[21] Xia X., Wang X., Cheng Z. (2019). The role of pyroptosis in cancer: pro-cancer or pro-"host"?. *Cell Death & Disease*.

[22] Li L., Jiang M., Qi L. (2021). Pyroptosis, a new bridge to tumor immunity. *Cancer Science*.

[23] Angell H., Galon J. (2013). From the immune contexture to the immunoscore: the role of prognostic and predictive immune markers in cancer. *Current Opinion in Immunology*.

[24] Gentles A. J., Newman A. M., Liu C. L. (2015). The prognostic landscape of genes and infiltrating immune cells across human cancers. *Nature Medicine*.

[25] Chae Y. K., Chang S., Ko T. (2018). Epithelial-mesenchymal transition (EMT) signature is inversely associated with T-cell infiltration in non-small cell lung cancer (NSCLC). *Scientific Reports*.

[26] Lu Y., Zhou X., Liu Z., Wang B., Wang W., Fu W. (2020). Assessment for risk status of colorectal cancer patients: a novel prediction model based on immune-related genes. *DNA and Cell Biology*.

[27] Suzuki K., Kachala S. S., Kadota K. (2011). Prognostic immune markers in non-small cell lung cancer. *Clinical Cancer Research*.

[28] Kobold S., Pantelyushin S., Rataj F., Berg J. v. (2018). Rationale for combining bispecific T cell activating antibodies with checkpoint blockade for cancer Therapy. *Frontiers in Oncology*.

[29] Popovic A., Jaffee E. M., Zaidi N. (2018). Emerging strategies for combination checkpoint modulators in cancer immunotherapy. *The Journal of Clinical Investigation*.

[30] Rizvi N. A., Hellmann M. D., Snyder A. (2015). Cancer immunology. Mutational landscape determines sensitivity to PD-1 blockade in non-small cell lung cancer. *Science*.

[31] Guo X., Zhang Y., Zheng L. (2018). Global characterization of T cells in non-small-cell lung cancer by single- cell sequencing. *Nature Medicine*.

[32] Fu D., Zhang B., Yang L., Huang S., Xin W. (2020). Development of an immune-related risk signature for predicting prognosis in lung squamous cell carcinoma. *Frontiers in Genetics*.

[33] Kayser G., Schulte-Uentrop L., Sienel W. (2012). Stromal CD4/CD25 positive T-cells are a strong and independent prognostic factor in non-small cell lung cancer patients, especially with adenocarcinomas. *Lung Cancer*.

[34] Bremnes R. M., Busund L.-T., Kilvær T. L. (2016). The role of tumor-infiltrating lymphocytes in development, progression, and prognosis of non-small cell lung cancer. *Journal of Thoracic Oncology*.

[35] Ma Q., Chen Y., Xiao F. (2021). A signature of estimate-stromal-immune score-based genes associated with the prognosis of lung adenocarcinoma. *Translational Lung Cancer Research*.

[36] Fridman W. H., Zitvogel L., Sautes-Fridman C., Kroemer G. (2017). The immune contexture in cancer prognosis and treatment. *Nature Reviews. Clinical Oncology*.

[37] Chen D. S., Mellman I. (2017). Elements of cancer immunity and the cancer-immune set point. *Nature*.

[38] Hegde P. S., Chen D. S. (2020). Top 10 challenges in cancer immunotherapy. *Immunity*.

[39] Faruki H., Mayhew G. M., Serody J. S., Hayes D. N., Perou C. M., Lai-Goldman M. (2017). Lung adenocarcinoma and squamous cell carcinoma gene expression subtypes demonstrate significant differences in tumor immune landscape. *Journal of Thoracic Oncology*.

[40] Langfelder P., Horvath S. (2008). WGCNA: an R package for weighted correlation network analysis. *BMC Bioinformatics*.

[41] Yu G., He Q. Y. (2016). ReactomePA: an R/Bioconductor package for reactome pathway analysis and visualization. *Molecular BioSystems*.

[42] Yu G., Wang L. G., Han Y., He Q. Y. (2012). clusterProfiler: an R package for comparing biological themes among gene clusters. *OMICS*.

[43] Wilkerson M. D., Hayes D. N. (2010). ConsensusClusterPlus: a class discovery tool with confidence assessments and item tracking. *Bioinformatics*.

[44] Yu J., Li S., Qi J. (2019). Cleavage of GSDME by caspase-3 determines lobaplatin-induced pyroptosis in colon cancer cells. *Cell Death & Disease*.

[45] Hu B., Elinav E., Huber S. (2010). Inflammation-induced tumorigenesis in the colon is regulated by caspase-1 and NLRC4. *Proceedings of the National Academy of Sciences of the United States of America*.

[46] Dunn J. H., Ellis L. Z., Fujita M. (2012). Inflammasomes as molecular mediators of inflammation and cancer: potential role in melanoma. *Cancer Letters*.

[47] Xi G., Gao J., Wan B. (2019). GSDMD is required for effector CD8^+^ T cell responses to lung cancer cells. *International Immunopharmacology*.

[48] Li Q., Chen L., Dong Z. (2019). Piperlongumine analogue L50377 induces pyroptosis via ROS mediated NF-*κ*B suppression in non-small-cell lung cancer. *Chemico-Biological Interactions*.

[49] Liu J., Yao L., Zhang M., Jiang J., Yang M., Wang Y. (2019). Downregulation of lncRNA-XIST inhibited development of non-small cell lung cancer by activating miR-335/SOD2/ROS signal pathway mediated pyroptotic cell death. *Aging (Albany NY)*.

[50] Zhou B., Zhang J. Y., Liu X. S. (2018). Tom20 senses iron-activated ROS signaling to promote melanoma cell pyroptosis. *Cell Research*.

[51] Wang Y., Shi P., Chen Q. (2019). Mitochondrial ROS promote macrophage pyroptosis by inducing GSDMD oxidation. *Journal of Molecular Cell Biology*.

[52] Goll D. E., Thompson V. F., Li H., Wei W., Cong J. (2003). The calpain system. *Physiological Reviews*.

[53] Zhang S., Deen S., Storr S. J. (2019). Calpain system protein expression and activity in ovarian cancer. *Journal of Cancer Research and Clinical Oncology*.

[54] Yu L. M., Zhu Y. S., Xu C. Z., Zhou L. L., Xue Z. X., Cai Z. Z. (2019). High calpain-1 expression predicts a poor clinical outcome and contributes to tumor progression in pancreatic cancer patients. *Clinical & Translational Oncology*.

[55] Al-Bahlani S. M., Al-Rashdi R. M., Kumar S., Al-Sinawi S. S., Al-Bahri M. A., Shalaby A. A. (2017). Calpain-1 expression in triple-negative breast cancer: a potential prognostic factor independent of the proliferative/apoptotic index. *BioMed Research International*.

[56] Xu F., Gu J., Lu C. (2019). Calpain-2 enhances non-small cell lung cancer progression and chemoresistance to paclitaxel via EGFR-pAKT pathway. *International Journal of Biological Sciences*.

[57] Gu J., Xu F. K., Zhao G. Y. (2015). Capn4 promotes non-small cell lung cancer progression via upregulation of matrix metalloproteinase 2. *Medical Oncology*.

[58] Lau J. K., Brown K. C., Dom A. M. (2014). Capsaicin induces apoptosis in human small cell lung cancer via the TRPV6 receptor and the calpain pathway. *Apoptosis*.

[59] Lee W. J., Shin C. H., Ji H. (2021). hnRNPK-regulated _LINC00263_ promotes malignant phenotypes through miR-147a/CAPN2. *Cell Death & Disease*.

[60] Chen J., Wu Y., Zhang L., Fang X., Hu X. (2019). Evidence for calpains in cancer metastasis. *Journal of Cellular Physiology*.

[61] Chen Y., Tang J., Lu T., Liu F. (2020). CAPN1 promotes malignant behavior and erlotinib resistance mediated by phosphorylation of c-Met and PIK3R2 via degrading PTPN1 in lung adenocarcinoma. *Thoracic Cancer*.

[62] Kang H.-G., Lee Y. H., Lee S. Y. (2021). Genetic variants in histone modification regions are associated with the prognosis of lung adenocarcinoma. *Scientific Reports*.

[63] Gorbunova A. S., Yapryntseva M. A., Denisenko T. V., Zhivotovsky B. (2020). BNIP3 in lung cancer: to kill or rescue?. *Cancers*.

[64] Sowter H. M., Ratcliffe P. J., Watson P., Greenberg A. H., Harris A. L. (2001). HIF-1-dependent regulation of hypoxic induction of the cell death factors BNIP3 and NIX in human tumors. *Cancer Research*.

[65] Glick D., Zhang W., Beaton M. (2012). BNip3 regulates mitochondrial function and lipid metabolism in the liver. *Molecular and Cellular Biology*.

[66] Gang H., Dhingra R., Lin J. (2015). PDK2-mediated alternative splicing switches Bnip3 from cell death to cell survival. *The Journal of Cell Biology*.

[67] Panigrahi D. P., Praharaj P. P., Bhol C. S. (2020). The emerging, multifaceted role of mitophagy in cancer and cancer therapeutics. *Seminars in Cancer Biology*.

[68] Zhang J., Ney P. A. (2009). Role of BNIP3 and NIX in cell death, autophagy, and mitophagy. *Cell Death and Differentiation*.

[69] Wang Y., Song X., Li Z. (2020). MicroRNA-103 protects coronary artery endothelial cells against H_2_O_2_-induced oxidative stress via BNIP3-mediated end-stage autophagy and antipyroptosis pathways. *Oxidative Medicine and Cellular Longevity*.

[70] Lu W., Wu Y., Huang S., Zhang D. (2021). A ferroptosis-related gene signature for predicting the prognosis and drug sensitivity of head and neck squamous cell carcinoma. *Frontiers in Genetics*.

[71] Chourasia A. H., Tracy K., Frankenberger C. (2015). Mitophagy defects arising from BNip3 loss promote mammary tumor progression to metastasis. *EMBO Reports*.

[72] Tormanen-Napankangas U., Soini Y., Kahlos K., Kinnula V., Paakko P. (2001). Expression of caspases-3, -6 and -8 and their relation to apoptosis in non- small cell lung carcinoma. *International Journal of Cancer*.

[73] Foveau B., Van Der Kraak L., Beauchemin N., Albrecht S., LeBlanc A. C. (2014). Inflammation-induced tumorigenesis in mouse colon is caspase-6 independent. *PLoS One*.

[74] Lin W., Chen Y., Wu B., Chen Y., Li Z. (2021). Identification of the pyroptosis‑related prognostic gene signature and the associated regulation axis in lung adenocarcinoma. *Cell Death Discovery*.

[75] Ye Y., Dai Q., Qi H. (2021). A novel defined pyroptosis-related gene signature for predicting the prognosis of ovarian cancer. *Cell Death Discovery*.

[76] Zheng M., Karki R., Vogel P., Kanneganti T. D. (2020). Caspase-6 is a key regulator of innate immunity, inflammasome activation, and host defense. *Cell*.

[77] Dagbay K. B., Hill M. E., Barrett E., Hardy J. A. (2017). Tumor-associated mutations in caspase-6 negatively impact catalytic efficiency. *Biochemistry*.

[78] Wang Q., Wang Y., Ding J. (2020). A bioorthogonal system reveals antitumour immune function of pyroptosis. *Nature*.

[79] Howrylak J. A., Nakahira K. (2017). Inflammasomes: key mediators of lung immunity. *Annual Review of Physiology*.

[80] Bergsbaken T., Fink S. L., Cookson B. T. (2009). Pyroptosis: host cell death and inflammation. *Nature Reviews. Microbiology*.

[81] Miao E. A., Leaf I. A., Treuting P. M. (2010). Caspase-1-induced pyroptosis is an innate immune effector mechanism against intracellular bacteria. *Nature Immunology*.

[82] Oh C., Verma A., Aachoui Y. (2020). Caspase-11 non-canonical inflammasomes in the lung. *Frontiers in Immunology*.

[83] Binnewies M., Roberts E. W., Kersten K. (2018). Understanding the tumor immune microenvironment (TIME) for effective therapy. *Nature Medicine*.

