# Supplementary material for: Pyroptosis-Related Gene Model Predicts Prognosis and Immune Microenvironment for Non-Small-Cell Lung Cancer
Source: Oxid Med Cell Longev. 2022 Aug 31;2022:1749111. doi: 10.1155/2022/1749111 (PMC9453043; doi:10.1155/2022/1749111)
Supplement: Supplementary Materials — Figure S1: immune infiltration supplements of prognostic genes. Figure S2: immunohistochemistry plots of prognostic genes from the HPA website. Figure S3: the relation between pyroptosis-related genes (PRGs) and immune. Table S1: univariate Cox regression analysis of PRGs in The Cancer Genome Atlas. Table S2: the survival situation in the risk groups in the training and testing cohorts. [file 1749111.f1.docx]

**Supplementary material**

**Figure S1**. Immune infiltration supplements of prognostic genes. (**A**, **C**, **E**) The correlation between six immune cells and the expression level of CAPN1 (**A**), BNIP3 (**C**), and CASP6 (**E**) in LUAD. (**B**, **D**, **F**) The correlation between six immune cells and the expression level of CAPN1 (**B**), BNIP3 (**D**), and CASP6 (**F**) in LUSC.

**Figure S2**. Immunohistochemistry plots of prognostic genes from the HPA website. (**A-C**) The plots of CAPN1 with the normal tissue (**A**), LUAD tissue (**B**), and LUSC tissue (**C**). (**D-F**) The plots of BNIP3 with the normal tissue (**D**), LUAD tissue (**E**), and LUSC tissue (**F**). (**G-I**) The plots of CASP6 with the normal tissue (**G**), LUAD tissue (**H**), and LUSC tissue (**I**).

**Figure S3.** The relation between pyroptosis-related genes (PRGs) and immune. (**A**) The expression level of PRGs in immune subgroups C1, cluster 1. C2, cluster 2. C3, cluster 3. (**B**, **D**, **G**) The correlation between the expression level of TIGIT and BNIP3 (**B**), CAPN1 (**D**), and CASP6 (**G**), respectively. (**C**, **E**, **H**) The correlation between the expression level of LAG3 and BNIP3 (**C**), CAPN1 (**E**), and CASP6 (**H**), respectively. (**F**) The expression level of immune checkpoints in two risk groups in the training cohort.

**Table S1.** Univariate Cox Regression Analysis of PRGs in The Cancer Genome Atlas. HR, hazard ratio; HR.95L, HR, 95H, hazard ratio 95% confidence interval.

**Table S2.** The survival situation in the risk groups in the training and testing cohorts.

**Figure S1**


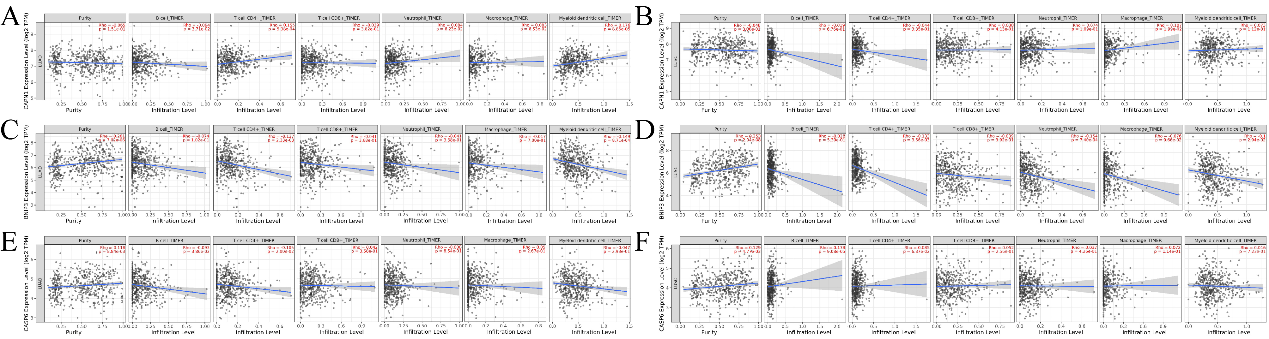


**Figure S2**


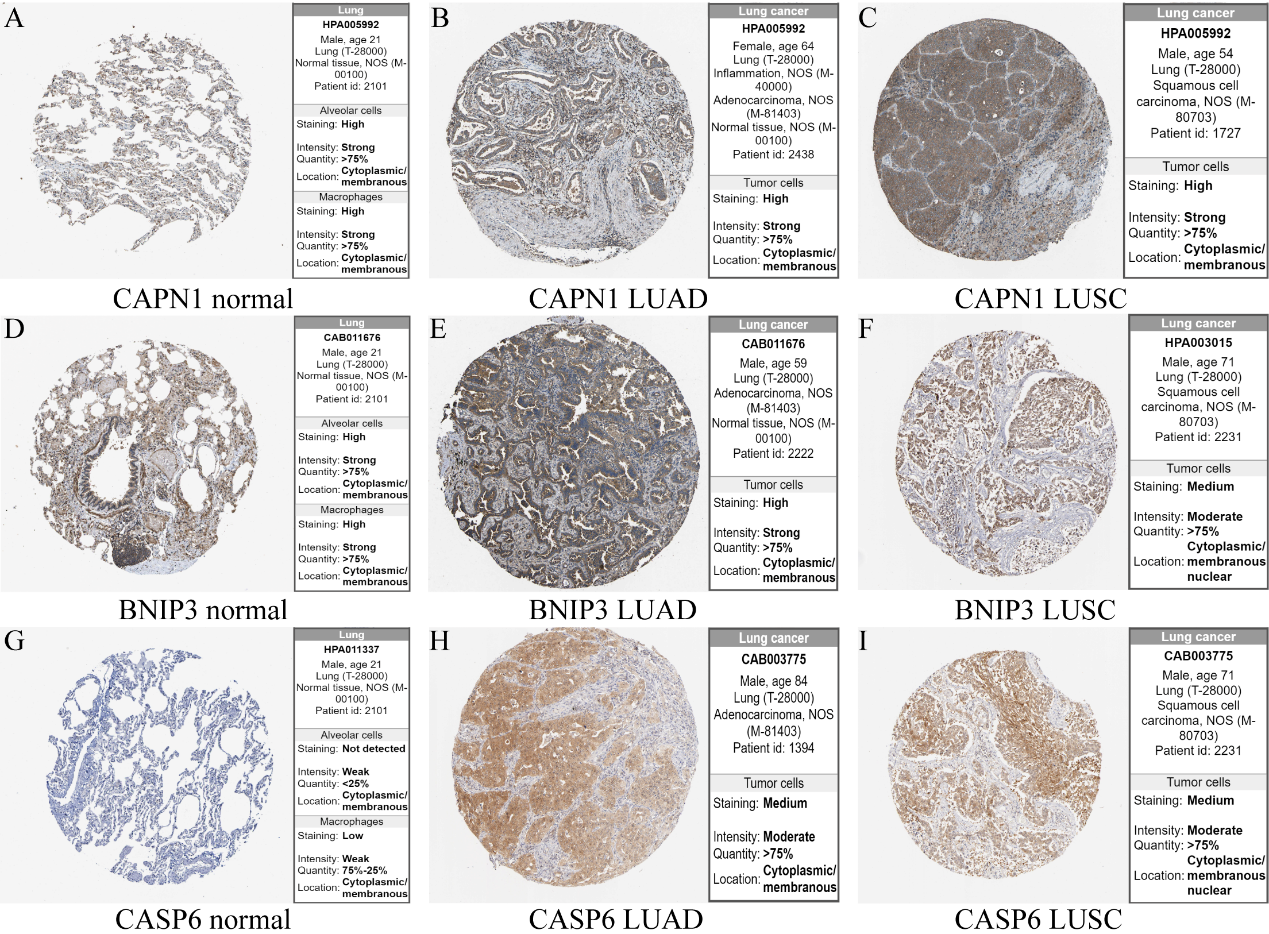


**Figure S3**


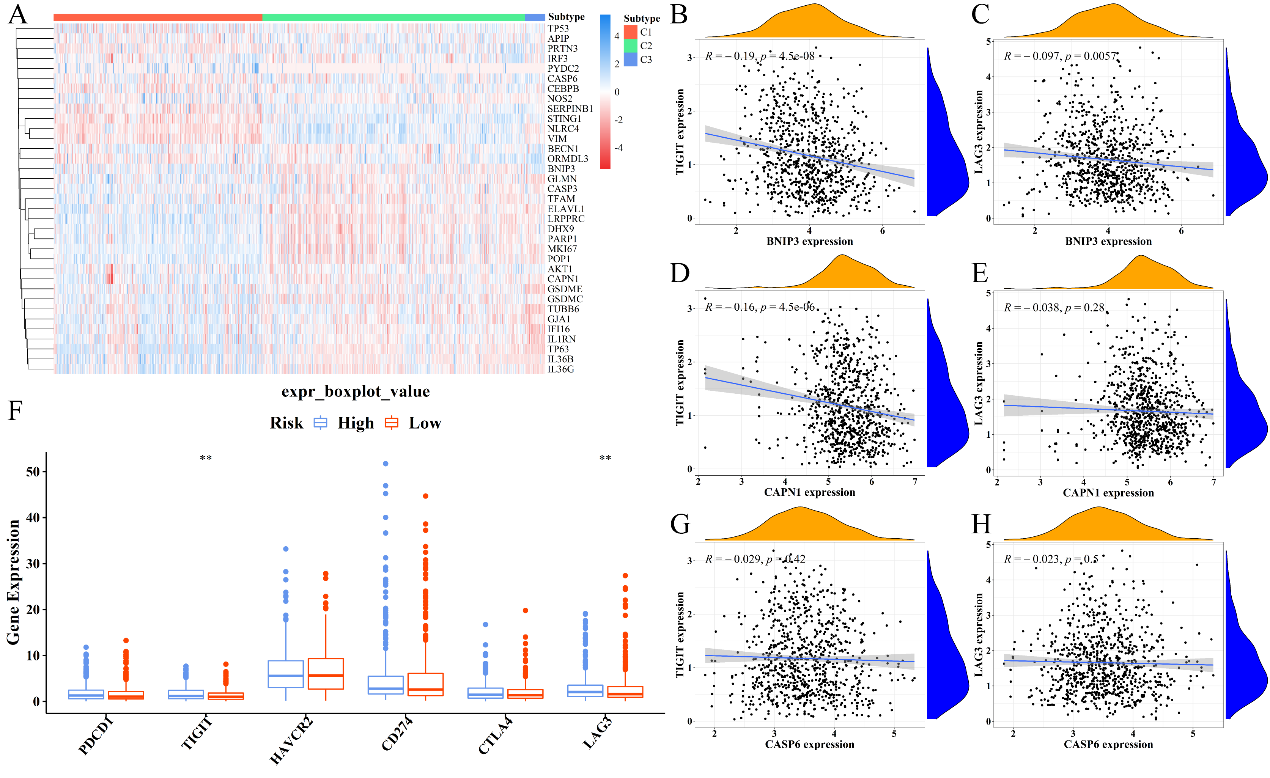


**Table S1.** Univariate Cox regression analysis of PRGs in The Cancer Genome Atlas.

| Gene | HR | HR.95L | HR.95H | p-value |
| --- | --- | --- | --- | --- |
| AKT1 | 1.003509 | 0.952973 | 1.056724 | 0.894308 |
| APIP | 1.037268 | 0.836328 | 1.286487 | 0.73909 |
| BECN1 | 0.972878 | 0.888163 | 1.065674 | 0.554152 |
| BNIP3 | 0.969151 | 0.940832 | 0.998321 | 0.038357 |
| CAPN1 | 1.017348 | 1.004251 | 1.030617 | 0.009279 |
| CASP3 | 1.011811 | 0.967986 | 1.05762 | 0.603245 |
| CASP6 | 0.935571 | 0.879594 | 0.99511 | 0.034371 |
| CEBPB | 0.996544 | 0.984431 | 1.008806 | 0.57901 |
| DHX9 | 1.00273 | 0.982839 | 1.023023 | 0.789719 |
| ELAVL1 | 1.062371 | 0.99989 | 1.128756 | 0.050418 |
| GJA1 | 1.000039 | 0.998194 | 1.001887 | 0.967168 |
| GLMN | 0.955141 | 0.806905 | 1.130609 | 0.593774 |
| GSDMC | 0.962964 | 0.913955 | 1.014601 | 0.156756 |
| GSDME | 1.04717 | 0.946994 | 1.157943 | 0.368977 |
| IFI16 | 1.005704 | 0.995033 | 1.01649 | 0.295958 |
| IL1RN | 1.004156 | 0.997027 | 1.011336 | 0.253925 |
| IL36B | 1.08635 | 0.950204 | 1.242003 | 0.225395 |
| IL36G | 1.002495 | 0.991943 | 1.013158 | 0.644428 |
| IRF3 | 1.000162 | 0.960909 | 1.041018 | 0.993676 |
| LRPPRC | 1.008237 | 0.976554 | 1.040948 | 0.614564 |
| MKI67 | 1.004719 | 0.957792 | 1.053946 | 0.847022 |
| NLRC4 | 0.910996 | 0.6246 | 1.328711 | 0.628335 |
| NOS2 | 0.994272 | 0.976364 | 1.012508 | 0.535584 |
| ORMDL3 | 0.993115 | 0.971201 | 1.015523 | 0.543935 |
| PARP1 | 1.005732 | 0.990734 | 1.020956 | 0.455932 |
| POP1 | 1.107516 | 0.966833 | 1.268671 | 0.140662 |
| PRTN3 | 1.266984 | 0.476391 | 3.369607 | 0.635384 |
| PYDC2 | 0.402943 | 0.004276 | 37.9718 | 0.695127 |
| SERPINB1 | 0.996181 | 0.989056 | 1.003358 | 0.296178 |
| STING1 | 0.991897 | 0.97382 | 1.01031 | 0.385959 |
| TFAM | 1.056595 | 0.988095 | 1.129844 | 0.107451 |
| TP53 | 1.00149 | 0.983621 | 1.019684 | 0.871212 |
| TP63 | 1.001231 | 0.997396 | 1.005081 | 0.529832 |
| TUBB6 | 1.00502 | 0.991907 | 1.018306 | 0.454888 |
| VIM | 1.000388 | 0.997907 | 1.002876 | 0.759358 |

**Abbreviations:** HR, hazard ratio; HR.95L, HR, 95H, hazard ratio 95% confidence interval.

**Table S2.** The survival situation in the risk groups in the training and testing cohorts.

| Cohort | Low-risk group | | | | High-risk group | | | |
| --- | --- | --- | --- | --- | --- | --- | --- | --- |
|  | Alive | Death | Total | Death rate | Alive | Death | Total | Death rate |
| Training cohort | 347 | 16 | 363 | 4.41% | 326 | 36 | 362 | 9.94% |
| Testing cohort | 150 | 168 | 318 | 52.83% | 58 | 88 | 146 | 60.27% |
| GSE37745 | 37 | 95 | 132 | 71.97% | 14 | 50 | 64 | 78.13% |
| GSE50081 | 74 | 48 | 122 | 39.34% | 32 | 27 | 59 | 45.76% |
| GSE102287 | 11 | 14 | 25 | 56.00% | 3 | 4 | 7 | 57.14% |
| GSE29013 | 28 | 11 | 39 | 28.21% | 9 | 7 | 16 | 43.75% |
